# Predictors of antiretroviral treatment failure to the first line therapy: a cross-sectional study among Iranian HIV-positive adults

**DOI:** 10.1186/s12879-024-09251-x

**Published:** 2024-03-28

**Authors:** Ava Hashempour, Nastaran Khodadad, Reza Ziaei, Behzad Rezaei, Farzaneh Ghasabi, Shahab Falahi, Azra Kenarkouhi, Mohammad Ali Davarpanah

**Affiliations:** 1 HIV/AIDS Research Center, Institute of Health, Shiraz University of Medical Sciences, Shiraz, Iran; 2 Zoonotic Diseases Research Center, Ilam University of Medical Sciences, Ilam, Iran; 3 Department of Microbiology, Faculty of Medicine, Ilam University of Medical Sciences, Ilam, Iran; 4 Department of Internal Medicine, Shiraz University of Medical Sciences, Shiraz, Iran

**Keywords:** Virological failure, Adherence level, HIV

## Abstract

**Background:**

HIV virological failure is one of the main problems in HIV-infected patients, and identifying the main predictors of such treatment failure may help in combating HIV/AIDS.

**Methodology:**

This cross-sectional study included 1800 HIV-infected patients with either virological failure or treatment response. HIV viral load, CD4 count, and other tests were performed. Statistical analysis was used to determine the predictors of virological failure.

**Results:**

Clinical stage, treatment with reverse transcriptase inhibitors (RTIs), under therapy for three years or more, suboptimal adherence to antiretroviral treatment (ART), age > 40 years, CD4 count < 200 cells/mm3, unemployment, being infected through sex, and the presence of symptoms were the predominant risk factors for virological failure. In addition, 55% of patients who experienced virological failure failed to experience immunological and/or clinical failure.

**Conclusion:**

As the first study in southern Iran and the second in Iran, Iranian policymakers should focus on intensive counseling and adherence support and emphasize more effective treatment regimens such as protease and integrase inhibitors (PIs and INTIs), to increase the chance of a treatment response to ART. The accuracy of identifying clinical and immunological criteria in resource-limited settings is not promising. The present findings can be used to determine effective measures to control HIV treatment failure and design efficient strategies for the ambitious 95–95–95 plan.

## Introduction

Infectious diseases with a broad range of clinical complications remain a noteworthy public health concern because they inflict numerous tragedies and great catastrophes in human history in different parts of the world [1–6]. Among various infections, HIV is a global crisis and a formidable challenge that has caused widespread epidemics in all countries, including Iran [7–9].

The predictors of virological failure have not been well studied in Iran; thus, this issue needs to be investigated in different populations. For example, in a report from Iran, only HIV-positive pediatric patients were studied; therefore, the results cannot be generalized to adult patients [10]. On the other hand, several reports have illustrated the presence of drug-resistance mutations that can lead to virological failure [7, 8, 11, 12]. As a result, addressing the factors that may cause treatment failure may clarify why the number of HIV-infected patients is increasing in Iran [13].

Zidovudine, a nucleoside reverse transcriptase inhibitor, was the pioneering therapy against HIV and gained FDA approval in 1987 [14]. By 1996, research demonstrated the benefits of combining medications for HIV treatment. This approach, known as ART, is recommended for all HIV patients by the World Health Organization (WHO) and the Department of Health and Human Services (DHHS) [15]. This particular course of treatment, which involves the daily consumption of multiple HIV medications, is known as an HIV regimen. An ordinary initial HIV regimen comprises three HIV medications from at least two different drug classes. Although it is not a curative measure, this treatment can significantly prolong the lives of patients and reduce the transmission of HIV [16].

The UNAIDS released a new set of ambitious targets, named the 95–95–95 plan, to control the global HIV epidemic by 2025, which should be followed at three levels: (1) 95% of all people infected with HIV should be diagnosed, (2) 95% of all individuals diagnosed with HIV infection should receive ART, and (3) 95% of all people under treatment should have an undetectable HIV viral load [17].

The 95–95–95 plan addresses inequalities in HIV treatment outcomes and outlines a vision for national AIDS responses. By closing gaps in testing and treatment coverage across countries, regions, and populations, we can achieve population-level declines in HIV incidence and ensure fairness in the global AIDS response [17]. The attainment of the 95-95-95 targets and the eventual eradication of the HIV/AIDS epidemic by 2030, following the global Sustainable Development Goals, can be greatly advanced through this approach. To meet the desired timeframe and ensure that no individual is overlooked, additional research is imperative in regions where progress has been limited [18].

Despite progressive achievements in preventing HIV/AIDS infection, treatment failure is one of the most significant predictors of mortality [19, 20].

In poor-resource countries, treatment failure can occur in three ways: immunological failure characterized by trends in CD4 counts, virological failure characterized by the measurement of HIV RNA levels, and clinical failure characterized by disease progression. Three types of failure may occur in combination or discordantly, as they do not occur according to expectations [21].

Since immunological and clinical failure criteria are inadequate for a definite diagnosis of treatment failure [22], the WHO recommended a viral load test as the confirmation approach for diagnosing HIV treatment failure [23]. Therefore, a prompt and accurate diagnosis of virological failure is essential to avoid additional side effects, drug resistance, treatment fatigue as a worldwide problem [24, 25], increased viral load, toxicity, high risk of failure against next-line agents, and increased risk of morbidity and mortality [26].

In low- and middle-income settings, the WHO recommends clinical and immunological monitoring to diagnose treatment failure, followed by an HIV viral load test to confirm treatment failure to avoid unnecessary ART regimen changes [27]. Since 2017, all HIV-positive patients have received free ART and relevant laboratory tests in Iran [28]; despite a global decline in the number of new HIV-positive patients, the incidence of HIV infection is increasing in Iran. Due to the costs, inaccessibility, and technical demands of the HIV RNA test, immunological and clinical criteria are the primary monitoring criteria used to diagnose treatment failure in Iran. However, clinical and immunologic parameters have unacceptable accuracy in identifying virologic failures, culminating in premature switching or continuous use of failed ART regimens and increasing morbidity and mortality rates [29]. Various factors can influence the response to viral infections [30–32].

To the best of our knowledge, only one report has assessed the predictors of treatment failure in Iran from only one city [33]. In the present study, a large area of Iran, which includes the southern provinces, was studied to gain deeper insight into identifying and intervening in determinants of virological treatment failure to control HIV transmission in Iran and achieve a 95–95–95 ambition plan.

## Materials and methods

### Study design, participants, and inclusion and exclusion criteria

The current study is a cross-sectional study that examined patients with HIV living in a large area of southern Iran living in Shiraz, Marvdasht, Darab, Larestan, Abadeh, Kazeroon, Jahrom, Fasa, Estahban, Bavanat, Darion, Yasuj, and Bushehr, which are located in Fars, Bushehr, and Kohgiluyeh, and Boyer-Ahmad provinces in Iran; these patients are registered at the Behavioral Diseases Counseling Center (BDCC) of Shiraz University of Medical Sciences to be treated with ART between 2020 and 2023. The clinical status, viral load, CD4 count, and biochemical and laboratory test results of these patients were recorded every six months. In addition, trained staff interviewed patients to collect various data regarding demographic characteristics, high-risk behaviors, etc.

In this center, almost 5000 patients were diagnosed, and only 1800 patients had an active file that followed their ART lines. Out of 1800 participants and considering the inclusion criteria, 132 patients with virologic failure were classified as the case group, and 132 patients without virologic failure were classified as the control group. These patients attended regular check-ups every 3 months at the center, and were interested in being part of the study. To be brief, the participants in the study consisted of patients who (1) were on RTIs and/or PIs (not integrase inhibitors) for ≥ six months and (2) had documented viral load measurements and CD4 cell counts at baseline and every six months. Not inclusion criteria included all participants with (1) TB-positive infection and (2) treatment interruption. In addition, due to their very small sample size and special clinical conditions, some patients, such as those who were younger than 18 years old and women with a pregnancy history of the past six months, were excluded from this study. Pregnant individuals require new antiretroviral medications and drug delivery methods that are both safe and efficient in managing HIV; unsurprisingly, this group is underrepresented in studies [34]. In addition, this investigation was carried out between 2020 and 2023, which coincided with the onset of the COVID-19 pandemic in Iran [35]. In other words, pregnant women preferred not to participate in the investigation because they might need to attend the center for unnecessary appointments, including collection of additional samples at a later time. Simultaneously, the center designed a strategy to minimize contact between pregnant women and staff due to various potential risks. Consequently, due to the very small number of expectant mothers, they were excluded from the investigation.

### Screening tests and immunological evaluations

The screening tests included rapid tests (SD Bioline HIV 1/2 3.0, Standard Diagnostics Inc. Kyonggi-do, South Korea) and the first (General Biologicals Corporation, Taiwan) and second (Dia. Pro, Italy) ELISA tests were performed for all referrals suspected of having HIV. If the results of all three tests were positive, the patient was treated.

### Sociodemographic and clinical data collection

Sociodemographic and clinical data, including the ART regimen, WHO clinical stage, baseline CD4 + T-cell count, initial regimen, adherence and duration of ART, and presence of any symptoms, were collected from patient medical records by an expert physician.

### Definition of terms

#### Baseline data

The data before ART initiation.

#### Virologic failure

In resource-limited countries, virological failure is defined as a viral load equal to 1000 copies/mL based on two consecutive viral load tests over 3 months, with adherence support during treatment [36, 37].

#### Immunological failure

Immunological failure is defined as (1) a decrease in the CD4 count to baseline or below or (2) persistent CD4 levels below 100 cells/mm3 for adults and adolescents [36].

#### Clinical failure

Clinical failure was defined as a new or recurrent clinical event indicating severe immunodeficiency (WHO clinical stage 4) after six months of treatment with good adherence [23].

### WHO clinical staging of HIV/AIDS

Based on symptoms in HIV-infected patients, the WHO classified HIV disease into four stages: I to IV (Table 1) [23].

**Table 1:** WHO clinical staging of HIV disease

Stage	Symptoms
**Clinical Stage 1**	Asymptomatic Persistent generalized lymphadenopathy
**Clinical Stage 2**	Moderate unexplained weight loss (< 10% of presumed or measured body weight) Recurrent respiratory tract infections (sinusitis, tonsillitis, otitis media, pharyngitis) Herpes zoster (in the last five years) Angular cheilitis Recurrent oral ulceration Popular pruritic eruption Fungal nail infections Seborrheic dermatitis
**Clinical Stage 3**	Unexplained severe weight loss (> 10% of presumed or measured body weight) Unexplained chronic diarrhea for longer than one month Unexplained persistent fever (intermittent or constant for longer than one month) Persistent oral candidiasis (now or in the last two years) Oral hairy leukoplakia Pulmonary tuberculosis (now or in the last two years) Severe bacterial infections (such as pneumonia, empyema, pyomyositis, bone or joint infection, meningitis, bacteremia) Acute necrotizing ulcerative stomatitis, gingivitis, or periodontitis Unexplained anemia (< 8 g/dl), neutropenia (< 0.5 × 10^9^/L) and/or chronic thrombocytopenia (< 50 × 10^9^/L)
**Clinical Stage 4**	HIV wasting syndrome *Pneumocystis (jirovecii)* pneumonia Recurrent severe bacterial pneumonia Chronic herpes simplex infection (orolabial, genital, or anorectal of more than one month in duration or visceral at any site) Esophageal candidiasis (or candidiasis of the trachea, bronchi, or lungs) Extrapulmonary tuberculosis Kaposi sarcoma Cytomegalovirus infection (retinitis or infection of other organs) Central nervous system toxoplasmosis HIV encephalopathy Extrapulmonary cryptococcosis, including meningitis Disseminated nontuberculous mycobacterial infection Progressive multifocal leukoencephalopathy (PML) Chronic cryptosporidiosis Chronic isosporiasis Disseminated mycosis (extrapulmonary histoplasmosis, coccidioidomycosis) Lymphoma (cerebral or B-cell non-Hodgkin) Symptomatic HIV-associated nephropathy or cardiomyopathy Recurrent septicemia (including nontyphoidal *Salmonella)* Invasive cervical carcinoma Atypical disseminated leishmaniasis

### Treatment failure

Treatment failure included clinical, immunological, and virological failure in those who were on ART. If a patient develops at least one of these three outcomes, they will be considered to have treatment failure [27]. In this study, case patients were defined as any individuals who experienced at least one of the treatment failure statuses.

### Responder patients

Patients who did not experience any virological, immunological, or clinical failure after six months of treatment. Here, the control group belonged to the abovementioned category and exhibited favorable virological, immunological, and clinical responses.

### Medication adherence

The rate of adherence refers to the prescribed dose of drug taken by the patient at a given time [5]. The adherence categories were considered good, unstable, or poor based on the self-report inventory. Patients with an excellent history of adherence were classified into the good adherence group, while those with intermittent phases of nonadherence or patients who rarely adhered to ARTs were classified into the unstable and poor adherence groups, respectively. The poor and unstable groups were defined as reduced adherence groups to compare the good adherence group with both the unstable and poor adherence groups [38].

### Specimen collection and processing

Expert laboratory staff collected 5 mL and 3 ml of whole blood into a plasma preparation tube (PPT) and anticoagulant ethylene diamine tetra-acetic acid (EDTA) tube for the participants’ viral load test, CD4 counts, and other laboratory tests. The tubes were centrifuged for 20 min at 3000 rpm to separate other parts of the blood samples. The collected specimens were labeled with study IDs and dates and transported directly to the laboratory for CD4 + T-cell count, the molecular laboratory for VL testing, and other laboratory experiments. The specimens were transferred to dry ice and stored at -80 °C until the tests were performed. Centrifugation, pipetting, and aliquoting were performed following standard protocols and laboratory biosafety precautions at the collection and testing sites.

### RNA extraction and HIV viral load

RNA was extracted from 0.5 mL of plasma using the QIAamp Viral RNA Mini Extraction Kit (Qiagen, Germany), and the viral load was subsequently measured using a quantitative real-time PCR (qRT‒PCR) HIV-1 assay with an Artus HI Virus-1 RT‒PCR kit (Qiagen, Germany).

### Determination of CD4 + T lymphocytes

The absolute counts of CD4 + T cells in whole blood specimens were quantified using a FACSPresto Near-Patient CD4 Counter (BD Biosciences, CA, USA). The CD4 + T-cell count was determined by adding 50 µl of whole blood sample to a tube containing 20 µl of monoclonal antibodies, followed by incubation for 30 min in the dark. The appropriate software identified the T lymphocyte populations and calculated the absolute counts of CD4 + cells.

### Biochemical, HCV, and HBV antibody tests

To assess the aminotransferase (ALT) (IU/L) and aspartate aminotransferase (AST) levels, 5 ml of fasting blood was collected, and the DIRUI Automatic Biochemistry Machine was used to measure the enzyme levels with commercial enzymatic kits from Biorex-Fars Company (Shiraz, Iran). In addition, commercially available ELISA kits were used for evaluating the seropositivity of specific antibodies against hepatitis B surface antigen (HBsAg, Dia. Pro., Italy) as well as HCV (HCVAb, Dia. Pro, Italy) according to the manufacturer’s protocols. The stop solution was used to terminate the enzymatic reaction. An ELISA-Reader (Epoch Microplate Spectrophotometer, Biotek, USA) was used to measure the absorbance, and the cutoff value was calculated according to the manufacturer’s instructions.

### Ethical consideration

The study was approved by the Ethical Review Committee of the Shiraz University of Medical Sciences, Shiraz, Iran. Written informed consent was obtained from each study participant, and those unwilling to contribute were excluded. The obtained samples and data were kept confidential using codes.

### Data processing and analysis

The data were checked for completeness and analyzed using SPSS version 20. The normality of the data was checked by the Shapiro‒Wilk test (*p* < 0.05). In addition, the homogeneity of group variances was tested by the Levene test. Depending on the normality of the data, either parametric or nonparametric tests were employed to compare the two studied groups.

Descriptive statistics are reported as the median, percentage, standard deviation (SD), mean, and interquartile range (Q1, Q3) for absolute and relative frequencies for categorical and continuous measures, including patient demographic, clinical, and treatment-related characteristics. In addition, chi-squared and Kruskal‒Wallis tests were used for appropriate variables and analyses. Independent variables with virological failure values less than 0.2 were analyzed by bivariant regression using a backward selection strategy. Logistic regression was used to examine the adjusted relationships between the study variables, including education, occupation, age, sex, marital status, drug use, TB coinfection status, disease stage, route of transmission, HCV and HBV coinfection status, etc., and outcome therapy.

Logistic regression model variables with odds ratios (ORs), 95% confidence intervals (CIs), and *P* values less than 0.05 were considered predictive factors for virological failure. Criteria from the STROBE guidelines were applied to evaluate the sampling method.

## Result

### Sociodemographic characteristics of the patients

In this research, two groups were analyzed—one consisting of 132 patients as cases and the other consisting of 132 patients as controls. The case group consisted of patients who experienced virological failure, with a viral load of 1000 copies/mL in two consecutive tests over a three-month period despite adherence support during treatment. The control group included individuals who did not have virological failure in two consecutive tests within a three-month period.

Based on the statistical power analysis results from G*Power (100%), the sample size was sufficient and the result can be generalized to the studied population. The majority of the patients in both groups were men (*n* = 204, 75%). While all patients suffered from virological failure, only 54% and 28% of patients experienced immunological and clinical failure, respectively. Compared to women, men exhibited greater clinical (77.4%) and immunological (54.5%) failure. Both poor and unstable adherence were rather high, accounting for 63.6% of all patients. According to the WHO classification, 86% of the patients were in stage I or II, and 14% were in stage III or IV. Only 31.8% of patients presented with one or more respiratory, gastrointestinal, neurological, or skin symptoms. Among various risk factors, including sexual contact, history of tattoo acquisition, etc., injecting drugs was the most prevalent risk factor in the history of the patients (%67). Concerning employment status, more than half of the patients (55.3%) were unemployed.

More details of the demographic and clinical factors are summarized in Table 2. The normality of the data was checked, and the data clearly supported that the distribution of the data significantly differed from a normal distribution.

**Table 2:** Patient demographic data and clinical characteristics

Parameters/Categories		Treatment Failure	Treatment Response	All Patients	*P* value
**Gender: female/male**		31/101 (24.3/76.5%)	32/100 (24.2/75.8%)	68/204 (25%/75%)	*p* value = 0.770
**Age, median (Min–Max)**		39 (18–65)	42 (18–69)	41 (18–69)	*p* value > 0.05
**Age range: 40≥**		36 (27.3%)	52 (39.4%)	88 (33.3%)	*p* value = 0.037
Educational status	Illiterate primary and secondary school Above secondary school	97/132 (73.5%) 35/132 (26.5%)	95/132 (72.7%) 72/132 (27.3%)	192/132 (72%) 107/132 (27%)	*p* value = 0.782
Occupation	Unemployed, including housewife Employed or specified job	81/132 (61.4%) 51/132 (38.6%)	65/132 (49.2%) 67/132 (50.8%)	146/132 (55.3%) 118/132 (44.7%)	*p* value = 0.048
Rout of HIV Transmission	IDU Sexual Mother-to-child transmission Others	68/132 (51.5%) 49/132 (37.5%) 15/132 (11.4%) 0/132 (0%)	111/132 (84.1%) 17/132 (125%) 3/132 (2.3.5%) 1/132 (0.8%)	183/264 (% 67.3) (%25) 68/264 19/264 (%7) (% 0.7) 2/264	*p* value > 0.001
AST (IU/L), median (Min–Max)		27 (4-200)	30 (9-325)	28 (4-200)	NA
ALT (IU/L), median (Min–Max)		24 (5-172)	29 (11–169)	28 (5-325)	NA
Liver Hepatotoxicity	No Yes	87.1% 12.6%	77.3% 22.7%	82.2 17.8	*p* value = 0.036
HCV Ab positive		71 (53.8%)	77 (58.3%)	148 (56.1)	*p* value = 0.457
HCV Ab and HBs Ag positive		(53.8%) 71	(70.5%) 93	164 (62.1%)	*p* value = 0.393
HIV viral load (log10copies/mL), median (Min–Max)		117,808 (1000-9.809e6)	NA	NA	NA
CD4 cell count (cells/mm2), median (Min–Max)		252 (8-1554)	545 (133–1726)	407 (8-1726)	*p* value = 0.282
CD4 cell count (cells/mm2)	> CD4 200 CD4 ≤ 200	(%55.1) 75 (%44.9) 61	(%96.3) 131 (%3.7) 5	(%75.7) 206 (%24.3) 66	*p* value > 0.001
Baseline CD4 cell count (cells/mm2), median (Min–Max)		216 (14-1489)	258 (12-1477)	230 (12-1489)	*p* value = 0.233
Years of HIV diagnosis, median (Min–Max)		10 (1–18)	8.5 (1–25)	9 (1–25)	*p* value = 0.374
Reverse Transcriptase inhibitor		105 (79.5%)	17 (12.9%)	122 (46.2%)	*p* value > 0.001
Treatment on ART (year), median (Min–Max)		6 (1–16)	6 (1–15)	6 (1–16)	*p* value > 0.05
Treatment on ART (year.) category	˂ 3 years ≤ 3 years = 5 years ≤ 5 years	7 (%5.3) 50 (%37.9) 75 (%56.8)	(%14.4) 19 (%28.8) 38 (% 56.8) 75	(%9.8) 26 (% 33.3) 88 (% 56.8) 150	*p* value = 0.028
Adherence category	Good Reduced	38/132 (28.8%) 94/132 (71.2%)	58/132 (43.9%) 74/132 (56.1%)	96/264 (36.4%) 168/264 (63.6%)	*p* value > 0.001
WHO clinical stage category	Stages 1 & 2 Stages 3 & 4	95 (72%) 37 (28%)	132 (100%) 0 (0%)	227 (86%) 37 (14%)	*p* value > 0.001
Symptom y/n (%) (gastrointestinal symptoms, respiratory symptoms, neurologic symptoms, and skin symptoms)		73/259 (55.3/44.7%)	11 (8.3%)	84/180 (31.8%/68.2)	*p* value > 0.001
Type of Treatment Failure	Virological F. Immunological F. Clinical F.	132 (100%) 42/132 (54%) 37/132 (28%)	NA	NA	NA
Immunological failure	In Female In Men	60/132 (45.5%) 72/132 (54.5%)	NA	NA	*p* value > 0.001
Clinical Failure	In Female In Men	37/132 (22.6%) 127/132 (77.4%)			*p* value > 0.001

a: Intravenous drug use

### Predictors of virological treatment failure

Using various tests, such as cross-tab tests, Mann–Whitney U tests, etc., only those with a value less than 0.2 were selected for binary regression, including those with a current CD4 + T-cell count less than 200, occupational level, reduced adherence to ART, WHO stages, etc. The results of the multivariable logistic regression model using forward conditional analysis revealed adjusted relationships between reduced adherence to ART, a CD4 + T-cell count less than 200, infection through sex, an RTI regimen, and age older than 40 years and virological failure (*p* value < 0.05) (Table 3).

**Table 3:** Multivariable logistic regression analysis for predictions of virological failure

Variables		Odd’s Ratio (OR)	Cl 95%			*p* value
			lower	upper		
Reverse Transcriptase inhibitor	NO	1				
	Yes	3.3	1.2		7.1	0.001
Infected through sex	NO					
	Yes	4.58	1.84		11.38	0.001
Adherence level	Good	1				
	Reduced	2.5	1.07		6.18	0.034
CD4 cell count (cells/mm2)	> CD4 200	1				
	CD4 ≤ 200	2.9	1.2		7.1	0.001
Gastrointestinal Symptom	No	1				
	Yes	3.2	1		10.6	0.05

## Discussion

Identifying and managing ART failure is one of the crucial challenges for controlling HIV infection at distinct levels, from virus transmission to mortality risk [26]. Although the 90-90-90 plan has not been fulfilled in Iran, active screening and case-finding strategies to register new HIV patients to start medical care are recommended as soon as possible to reduce the chance of treatment failure. In other words, there is a prompt need to identify sustainable treatment strategies and prevention measures to reduce HIV treatment failure, HIV transmission, and the incidence of new HIV cases.

In this study, it was one of the important indicators of treatment failure. There was a significant difference between the frequency of reduced adherence in the case (71.2%) and control (56.1%) groups. In addition, logistic regression analysis revealed that the probability of virological failure in patients with reduced adherence was 2.5 times greater than that in patients with good adherence (OR = 2.5, *P* value = 0.045), which was in line with some reports indicating that good adherence to ART may prevent treatment failure [39–41].

Based on several reports, low adherence to ART reduces viral suppression, which consequently contributes to drug resistance and treatment failure, increasing the risk of patient vulnerability to opportunistic infections and even mortality [42, 43]. To improve adherence to ART, Iranian policymakers have provided it free of charge for all HIV-infected patients as soon as a diagnosis since 2017 [28]. However, due to some limitations, there is no effective policy to improve patients’ adherence levels in Iran, which has led to an increase in HIV transmission, which has a detrimental influence on public health. It is worth mentioning that the development of patient-centered strategies, the simplification of regimen-specific obstacles such as minimizing pill burden through fixed-dose combination (FDC), once-daily complete regimens, medications that can be taken without food, individualized patient training, adherence evaluation, and adherence support plans, etc., will improve quality of life, benefit long-term medication adherence and lessen the risk of treatment failure.

For years, RTIs have been widely used to suppress HIV viral replication; however, our data suggested that patients receiving RTIs were 3.3 times more likely to face treatment failure than those receiving RTIs and PIs (OR = 3.3, *P* = 0.001). This result was in accordance with other studies that indicated that patients treated with RTIs were more likely to face treatment failure since RTIs are a low genetic barrier to resistance, and even single-point mutations in HIV can be responsible for the loss of drug susceptibility. In contrast, PIs are classified as having a high genetic barrier to resistance and continue to function even after several mutations in HIV genomes. Therefore, patients treated with PIs usually experience treatment failure, and the immune system can be efficiently restored [44]. According to the US Agency for International Development, 46% of HIV-infected patients who fail RTIs have a greater chance of failing PIs [45, 46]; furthermore, PIs have narrow choices for further switching, a primary concern in resource-limited settings [22]. Thus, individuals treated with RTIs need more specialist support and even intervention to adhere to their treatment plan.

Here, logistic regression revealed that compared with infection through injection, infection via a sexually infected agent increased the risk of virological failure by 4.8 times. (OR = 4.85, *P* < 0.001). Based on previous studies, contrary to HIV transmission through injections, a history of unprotected sexual contact significantly resulted in treatment failure since sexual exposure to HIV increased the risk of transmission of a diverse HIV population compared to other HIV-transmitted methods [47, 48].

IDUs were found to experience a slower progression of HIV disease than those who were exposed to the virus through sexual transmission, likely due to the varying target cells that HIV impacts. For example, sexually transmitted HIV first infects antigen-presenting Langerhans cells and subsequently infects other cells, while intravenously transmitted virus promptly targets lymphocytes [49]. Therefore, setting policies to control sexual HIV transmission is highly necessary to reduce the rate of treatment failure among the Iranian population.

The other factor related to virological failure is the CD4 count, the backbone of the immune system, which protects the body from diverse diseases and can prevent HIV replication [36]. The CD4 + cell count reflects the status of the immune system; thus, a reduction in CD4 + T lymphocyte counts predisposes patients to opportunistic infections [50]. According to our data, patients with CD4 < 200 cells/mm were 41 times more likely to experience virological failure than were those with CD4 ≤ 200 cells/mm (OR = 41, *P* < 0.001). In line with our findings, another study reported that the probability of viral treatment failure is nine times greater in these patients than in those with higher CD4 T-cell counts [51–54]. A high level of HIV replication indicates a decreased CD4 + T-cell count and poor immune responses, leading to virological failure [55].

The median age in both groups was approximately 40 years, indicating that the subjects were middle aged. Patients older than 40 years had a 3.3 times greater chance of treatment failure than those younger than 40 years (*P* = 0.041), which was in accordance with some reports [41, 51]. In another report, older age was associated with lower CD4 + counts, advanced stages of HIV disease, bedridden functional status, and delayed diagnosis of HIV/AIDS [56]. In another study, adverse clinical outcomes, depression, a lower status of disclosure, and social isolation were reported in older patients with HIV-positive status. The elderly population necessitates an early diagnosis since the CD4 cells in these individuals are present at a diminished level, consequently resulting in a more compromised immune system and less efficient response to ART [33, 34].

In our opinion, those who received ART for three years or more were at significant risk of virological failure (*P* = 0.028), which is consistent with the findings of another study suggesting that prolonged ART use caused a detectable HIV viral load [57]. Moreover, a study of the Bale Zone declared that older ART clients experienced 2.91-fold greater treatment failure [58]. Patients who take ART for a longer time may develop treatment failure considering the deficit in immune reconstitution functions, which gradually increases with reduced adherence, age, and an increase in the incidence of complications or side effects of the treatment.

Additionally, patients with gastrointestinal, neurologic, respiratory, and skin symptoms significantly experienced virological failure (*P* = 0.001), which agreed with the findings of a study from Switzerland showing that respiratory signs increase the chance of virological failure [52].

The incidence of treatment failure in HIV-infected individuals with advanced WHO stage III or IV disease was greater than that in patients with stage I or II disease (*P* = 0.001). These data align with data collected in Malawi, northwestern Ethiopia, and sub-Saharan Africa [59]. Patients categorized as WHO clinical stage IV are likely to be infected with opportunistic microorganisms, and the drugs prescribed to treat opportunistic infection may cause drug interactions with ART that minimize the potency of ART, leading to a higher rate of treatment failure [27]. Therefore, early diagnosis and prompt initiation of ART delay disease progression.

Our data revealed that education level did not correspond to treatment failure, which was consistent with the findings of Ayele et al. in Ethiopia [24] but was in line with the findings of other studies [46, 60]. The difference may be due to the study participants’ awareness of treatment failure, as HIV-infected patients in other studies [46, 60] mostly came from urban areas (79%). In contrast, most of our study participants lived in urban areas.

Moreover, the possible role of unemployment in treatment failure was found among our patients, which showed that unemployment may reduce treatment adherence. A job is a valid characteristic of an individual’s socioeconomic status in society; therefore, unemployment is a risk factor for the incidence of treatment failure [61].

In this study, the frequency of immunological and clinical failure did not differ between males and females in China and those in other countries. This may be related to similar CD4 counts in females and males during ART initiation.

Furthermore, coinfection with HBV and/or HCV did not affect the treatment outcome. However, some studies have shown that the probability of virological failure among patients infected with HBV is slightly lower than that among HBV-negative individuals. One may assume that some RTIs are effective against both HBV and HIV [62]; moreover, HBV treatment may also cause HIV suppression [63, 64]. There was no significant difference between the rate of HCV Ab in the two groups of patients who experienced treatment failure (53.8%) or who experienced a response to treatment (58.3%) (*p* < 0.05) since the rate of HCV Ab positivity was high in both groups. However, it was proposed that the effective treatment of HCV lowers the treatment failure rate in patients infected with HIV and HCV to prevent immune system weakness and treatment failure [65, 66].

According to our data, 17.8% (47/264) of patients experienced liver hepatotoxicity, as measured by the ALT level, which agreed with the findings of other studies [67, 68]. Liver enzyme elevation commonly represents possible liver injury, a crucial step in managing HIV-infected individuals. HIV infection and ART can hurt hepatocytes directly and indirectly and may impact the pathogenesis of liver disease. Here, the incidence of hepatotoxicity was significantly lower in the treatment-responsive patients (*P* value = 0.039). Distinguishing drug-induced hepatotoxicity from the hepatic damage induced by viral infections is complex and challenging [69]. The adherence level in the responder group of patients was significantly greater than that in the treatment failure group, suggesting that despite the benefits of the ART regimen, it may hurt hepatocytes, increasing the risk of hepatotoxicity, which highlights the need for a national program comprising the periodic monitoring of hepatotoxicity screening tests in patients with HIV as part of comprehensive care for HIV-infected patients.

In the present report, all patients suffered from virological failure, while only 54% and 28% experienced immunological and clinical failure, respectively. A possible explanation for this contradiction may be that clinical and immunological criteria have relatively low or poor sensitivity and have a positive predictive value for determining HIV treatment failure [70].

Thus, viral load-based treatment failure may be regarded as a more efficient prognostic method to predict clinical and immunological failure as well as advanced stages of HIV infection, which is beneficial for improving treatment. Therefore, viral load monitoring has become the standard for monitoring the outcome of ART; however, HIV-1 RNA above the cutoff has been chosen as a public health approach to minimize unnecessary switching to more burdensome and costly HIV regimens [26].

Overall, the diversity observed in the present study may be related to the number of enrolled participants, lifestyle factors, diverse demographics, population genetics, and various study failure assessment criteria.

Despite our best efforts to estimate virological failure and its predictors, there are certain limitations that we must acknowledge. The cross-sectional nature of our study prevented us from including all possible factors that could impact the incidence of virological failure. For instance, we did not evaluate parameters such as the hemoglobin level or adverse effects of ART. Furthermore, psychosocial and emotional factors, including anxiety, depression, and stigma, were not investigated. Another significant constraint of this study was that, similar to several other illnesses, the HIV/AIDS detection rate in Iran is not conveniently high [71], which affects the percentage of treatment failures. Furthermore, we encountered the obstacle of not being able to incorporate one of the HIV exposure categories. These men have sex with men due to their unavailability and because such data are not typically gathered in the routine surveillance system in Iran. However, this may not pose a problem, as the two primary routes of HIV transmission in Iran are through injection drug use and sexual contact.

Some suggestions can be considered in future studies. Although this study was conducted in patients living in large areas of Iran, the factors affecting the treatment response rate may not be generalizable to all Iranian HIV-infected patients. Therefore, further study is needed periodically in the broader Iranian population to define whether there are differences in clinical, immunological, and virological responses to ART. In addition, studies can be designed to determine the effect of the support of the health care system on the adherence of patients with poor adherence to ART. Overall, various measures can be taken in Iran to support and train high-risk people to control high-risk sexual behavior, which can reduce treatment failure and HIV spread in Iran.

## Conclusion

The present study showed that the following conditions were the most important predictors of treatment failure: a CD4 + lymphocyte count less than 200 cells/ml, reduced adherence to ART, advanced WHO stage, treatment with only RTIs, infection through sexual relationships, presence of symptoms, age > 40 years, duration of ART, unemployment, and treatment with ART for three years or more. The factors mentioned above should be considered when characterizing patients with a greater risk of treatment failure. In other words, these data emphasize that a reduction in treatment failure could easily be achieved by considering protective factors, including early diagnosis, good adherence to ART, and immediate ART treatment.

Clinical and immunologic treatment failure criteria missed 55% of the treatment failure discovered by viral load testing, indicating that clinical and immunological criteria are ineffective in predicting ART virological failure. Early identification of treatment failure gives patients a greater chance of success in response to therapy when switching to a second-line ART.

Therefore, the current strategies should be revised to achieve improved drug adherence, rigorous clinical follow-up, timely diagnosis of individuals with HIV, prompt initiation of assisted reproductive technology (ART), and viral load monitoring for HIV-infected patients in Iran, all of which can help to increase the effectiveness of first-line therapy. To monitor the progress of the national action plan of 95–95–95 strategies, the predictors of virological failure among adult patients can help healthcare professionals and policymakers plan and design appropriate intervention strategies, enabling the country to sustain successes that would assist in minimizing mortality among ART clients at the national level by enhancing treatment success.

## Data Availability

All the data can be obtained upon request from the corresponding author and the first author (Ava Hashempour, email address: thashem@sums.ac.ir).
